# Commensal *Hafnia alvei* strain reduces food intake and fat mass in obese mice—a new potential probiotic for appetite and body weight management

**DOI:** 10.1038/s41366-019-0515-9

**Published:** 2020-01-07

**Authors:** Romain Legrand, Nicolas Lucas, Manon Dominique, Saida Azhar, Camille Deroissart, Marie-Anne Le Solliec, Julie Rondeaux, Séverine Nobis, Charlène Guérin, Fatima Léon, Jean-Claude do Rego, Nicolas Pons, Emmanuelle Le Chatelier, S. Dusko Ehrlich, Gregory Lambert, Pierre Déchelotte, Sergueï O. Fetissov

**Affiliations:** 1 TargEDys SA, University of Rouen, 76183 Rouen, France; 2 Inserm UMR1073, Nutrition, Gut and Brain Laboratory, 76183 Rouen, France; 3 Institute for Research and Innovation in Biomedicine (IRIB), University of Rouen Normandy, 76183 Rouen, France; 4 Animal Behavioral Platform SCAC, University of Rouen Normandy, 76183 Rouen, France; 5 MGP MetaGénoPolis, INRA, Université Paris-Saclay, 78350 Jouy en Josas, France; 6 Rouen University Hospital, CHU Charles-Nicolle, 76183 Rouen, France; 7 Laboratory of Neuronal and Neuroendocrine Differentiation and Communication, Inserm UMR1239, Mont-Saint-Aignan, France

**Keywords:** Obesity, Obesity, Bacteria

## Abstract

**Background/objectives:**

Based on the recent identification of *E.coli* heat shock protein ClpB as a mimetic of the anorexigenic α-melanocyte stimulating hormone (α-MSH), the objective of this study was to preclinically validate *Hafnia alvei*, a ClpB-producing commensal bacterium as a potential probiotic for appetite and body weight management in overweight and obesity.

**Methods:**

The involvement of enterobacterial ClpB in the putative anti-obesity effects was studied using ClpB-deficient *E.coli*. A food-grade *H. alvei* HA4597 strain synthetizing the ClpB protein with an α-MSH-like motif was selected as a candidate probiotic to be tested in *ob/ob* and high-fat diet (HFD)-fed obese and overweight mice. The relevance of the enterobacterial ClpB gene to human obesity was studied by in silico analysis of fecal metagenomes of 569 healthy individuals from the “MetaHIT” database.

**Results:**

Chronic per os administration of native but not ClpB-deficient *E.coli* strain reduced body weight gain (*p* < 0.05) and daily meal frequency (*p* < 0.001) in *ob/ob* mice. Oral gavage of *H.alvei* for 18 and 46 days in *ob/ob* and HFD-fed obese mice, respectively, was well tolerated, reduced body weight gain and fat mass in both obesity models (*p* < 0.05) and decreased food intake in hyperphagic *ob/ob* mice (*p* < 0.001). Elevated fat tissue levels of phosphorylated hormone-sensitive lipase were detected in *H.alvei* -treated *ob/ob* mice (*p* < 0.01). Enterobacterial ClpB gene richness was lower in obese vs. non-obese humans (p < 0.0001) and correlated negatively with BMI in genera of *Enterobacter, Klebsiella* and *Hafnia*.

**Conclusions:**

*H.alvei* HA4597 strain reduces food intake, body weight and fat mass gain in hyperphagic and obese mice. These data combined with low enterobacterial ClpB gene abundance in the microbiota of obese humans provide the rationale for using *H.alvei* as a probiotic for appetite and body weight management in overweight and obesity.

## Introduction

The etiology of obesity is multifactorial, with the genetic predisposition playing a key role including both the human genome and the metagenomes of multiple microorganisms constituting the body microbiota inhabiting various epithelial surfaces [1]. The gut microbiota is the principal source of bacteria, viruses and fungi in the body with the number of bacteria approximately equal to the total number of human cells [2]. The composition of the gut microbiota changes rapidly due to diet and is influenced by age, sex, geographical location and drugs such as antibiotics [3, 4]. The altered composition of the gut microbiota in obesity has been well documented; its transfer in experimental animals was accompanied by the transfer of an obesity phenotype providing proof of its causal role in obesity pathogenesis [5, 6]. Inversely, a healthy microbiota contribute to the regulation of various physiological processes including the regulation of feeding behavior [7].

It is hence conceivable that the restoration of a healthy microbiota or a modulation of its composition aimed at increasing beneficial bacterial species may represent a strategy for appetite and body weight management in obesity [8]. However, several recent papers reviewed the limited efficiency of traditional probiotic bacteria of the *Lactobacillus* and *Bifidobacterium* species in obesity [9, 10]. A new generation of probiotics should be developed based on the analysis of the gut microbiota composition and a better understanding of the mechanisms of action of commensal bacteria on the host [11]. In this study, we used the recently generated data of specific bacteria-host communication to develop a new potential probiotic for appetite and body weight management in obesity.

The key underlying finding was the identification of *E.coli* heat shock protein ClpB as an antigen-mimetic of the anorexigenic α-melanocyte stimulating hormone (α-MSH) [12]. Unexpectedly, the same study showed that oral gavage to lean mice with *E.coli* native but not ClpB-deficient strains decreased their food intake and body weight, suggesting a key role of the ClpB in the anorexigenic effect of *E.coli*. Considering the key role of α-MSH-mediated melanocortin signaling system in the regulation of energy balance, exemplified by marked hyperphagia and obesity in its deficient states in both rodents and humans, the α-MSH mimetic properties of bacterial ClpB suggest that commensal bacteria producing similar with the *E.coli* ClpB protein, may be used as an anti-obesity probiotic [13–15].

Thus, the objectives of this study were to demonstrate the relevance of ClpB protein in potential anti-obesity effects of ClpB expressing bacteria and to preclinically validate *Hafnia alvei* (*H.alvei*) a food-grade, commensal specie of the *Hafniaceae* (formerly *Enterobacteriaceae*) family as a putative anti-obesity probiotic [16]. For this purpose, the presence of α-MSH mimetic epitopes in the ClpB protein of the *H.alvei* HA4597 strain was analyzed using both in silico and proteomic approaches. Then, we tested *H.alvei* HA4597 in two mouse models of obesity: genetic, leptin-deficient *ob/ob* mice and nutritional, high-fat diet (HFD)-induced obesity. The complementarity of these models is related to the hyperphagia and severe obesity with a standard chow consumption in *ob/ob* mice combined with moderate obesity in otherwise normo-/hypophagic HFD-fed mice as a model of nutritionally induced overweight. In addition, to test the relevance of ClpB to the anti-obesity effects, in a separate experiment, ClpB-expressing and ClpB-deficient bacteria were supplied to *ob/ob* mice. Finally, to further justify the rationale for supplementation of ClpB-expressing probiotic in humans, we performed in silico analysis of the metagenomes from the human fecal microbiota samples of 569 healthy individuals available from the database of the MetaHIT consortium [17] and in which the prevalence of the *E.coli* ClpB gene was analyzed in relation to BMI and obesity.

## Materials and methods

### Animals

Animal experiments were approved by the Local Ethical Committee of Normandy (approval N5986). All mice were purchased from Janvier Labs (L’Arbresle, France); they were housed in a specialized animal facility (22 ± 2 °C, relative humidity 40 ± 20%) under a 12 h light (7:00 a.m.–7:00 p.m.)/12 h dark cycle. Mice were kept in standard plastic cages (*n* = 3 per cage) with ad libitum food (3430 Kliba Nafag standard diet, Kaiseraugst, Switzerland, unless exposed to an HFD) and water access. B6.V-Lep *ob/ob* JRj mice (*n* = 75) and C57 Bl/6 JRj mice (*n* = 70) both males were 6 weeks of age at arrival. After acclimation for 7 days, C57Bl/6 mice had *ad libitum* access to an HFD with the following caloric content: 45% fat, 35% carbohydrates, and 20% proteins (D12451, Research Diets, New Brunswick, NJ, USA) for 19 weeks. Food intake per cage and individual body weight were measured daily. For the study of the effects of ClpB-deficient *E.coli* strain on feeding behavior, after 7 days of acclimation *ob/ob* mice were placed in individual BioDAQ cages (Research Diets).

### Experimental procedures in mice

For the study of the ClpB-deficient *E.coli* K12 effects, *ob/ob* mice were randomly divided into three groups (*n* = 8 of each) with the same mean body weight. Sample size was estimated based on previous experiments [12]. One group received the *E.coli* K12 native strain and another group the *E.coli* K12 ClpB-deficient strain; both strains were received in LB medium and the control group received LB medium only via intragastric gavage as described below for *H.alvei*. The experimental procedure was alike the one described above but mice were kept in individual BioDAQ cages. The *E.coli* strains and culture conditions have been previously described [12].

For the study of *H.alvei* HA4597 effects, both *ob/ob* and C57Bl6 mice with obesity induced by HFD were randomly divided into two groups *ob/ob* (*n* = 12, in each) and HFD (*n* = 22 in each) with the same mean body weight. Randomization was performed by each cage of three so that the animals from the same cage belonged to the same group. From day 1 onwards, mice received once daily at 6:00 p.m. *H.alvei* HA4597 cultured strain in LB medium or LB medium (control group) in a volume of 10 mL/kg, via intragastric gavage using a steel drenching cannula (Socorex, Ecublens, Switzerland). Before the gavage, individual body weight and food & water intakes (by cage) were measured. Abnormal symptoms and behavior (sickness and aggressiveness) were monitored and if they occurred, such animals were removed from the study. Body composition was analyzed using the MiniSpec LF50 (Bruker, Rheinstetten, Germany) before and at the end of the treatment (day 18 for *ob/ob* and day 46 for HFD mice). Then, mice were terminally anesthetized by intraperitoneal injection of ketamine/xylazine solution (80/10 mg/kg) and a blood sample (~0.5 mL) was taken via abdominal aorta puncture in aprotinin EDTA (1 mg/mL) containing tubes (BD Vacutainer, Franklin Lakes, NJ, USA). The tubes were centrifuged at 1500 × *g* during 20 min, and plasma samples were stored at −80 °C. The following tissues were dissected and snap-frozen in liquid nitrogen prior to storage at −80 °C: hypothalamus, epididymal fat pads, and fecal colon content.

### Proteomic analysis of Hafnia alvei HA4597

The TargEDys proprietary *H.alvei* HA4597 strain (manufactured by Biodis, Noyant, France) was analyzed for the presence of α-MSH-like epitopes using western blot (WB) and immunoprecipitation with α-MSH antibodies (Delphi Genetic, Charleroi, Belgium). Total protein from *H.alvei* HA4597 cultures was extracted and processed for WB. Shotgun LC-MS/MS mass spectrometry (Biognosys, Schlieren, Switzerland) was used for the identification of bacterial proteins precipitated by α-MSH antibodies after total protein extraction from *H.alvei* HA4597 and *E.coli* K12. For the detailed procedure see Supplementary data.

### Hafnia alvei HA4597 strain preparation for animal experiments

The *H.alvei* HA4597 strain was pre-cultured by incubating under agitation of 100 µL of the bacterial stock suspension in 10 mL of Luria Broth culture medium (LB, Conda, Madrid, Spain) overnight at 37 °C. The optical density of diluted preculture (1:10 vol. in culture medium) was measured using a spectrophotometer at 600 nm (LB medium served as blank) to adjust the optical density at 0.01 at the beginning of the culture. Then, the bacterial culture was started with LB medium at 37 °C under agitation. After 4 h of incubation, *H.alvei* culture samples were taken and stored at −20 °C until use. The number of CFU per animal was determined prior to the gavage consisting of 3 × 10^8^ CFU/day for *ob/ob* mice and 4 × 10^7^ CFU/day for the HFD model.

### ClpB protein assay

Development and validation of an *E.coli* ClpB immunoassay has been described [18]. In brief, rabbit polyclonal anti-*E.coli* K12 ClpB antibodies (Delphi Genetics) were coated onto 96-well Maxisorp plates (Nunc, Rochester, NY). One hundred microliters of plasma samples or ClpB protein (Delphi Genetics) standard dilutions were incubated for 2 h at room temperature (RT). Mouse monoclonal anti-*E.coli* K12 ClpB antibodies (Delphi Genetics) and goat anti-mouse alkaline phosphatase conjugated IgG (Jackson ImmunoResearch Laboratories Inc., West Grove, USA) were used for ClpB detection and p-nitrophenyl phosphate solution (Sigma-Aldrich, St. Louis, USA) as a substrate. The reaction was stopped by 3 N NaOH and optical density was determined at 405 nm using a microplate reader Metertech 960 (Metertech Inc., Taipei, Taiwan). Concentration was measured by referring to the ClpB protein standard curve.

### ClpB DNA in fecal content

Total DNA was extracted from the colonic fecal content using the QIAamp Mini Spin Columns, following the manufacturer’s instructions (Qiagen, Courtaboeuf, France). The PCR conditions and ClpB DNA primers have been described [12], 16 S rRNA gene primers were used from Turner et al. [19].

### Phosphorylated hormone-sensitive lipase (pHSL) levels in fat tissue

To evaluate the possible effects of *H.alvei* HA4597 supplementation on fat tissue lipolysis, the levels of pHSL protein, a lipolytic marker, were studied by WB in the epididymal fat tissue of both models using anti-pHSL antibodies (Cell Signaling Technology, MA, USA).

### Expression of hypothalamic neuropeptides

To study the possible effects of H.alvei HA4597 supplementation on the hypothalamic feeding-regulatory neuropeptides, mRNA expression of agouti-related protein (AgRP), neuropeptide Y (NPY), and proopiomelanocortin (POMC) were determined by RT-PCR. Total RNA was extracted from mouse hypothalami in TRIZOL reagent according to the supplier instructions (Invitrogen, Carlsbad, CA, USA). RT-PCR was performed using 1 µg of total RNA and 200 U of SuperScript II reverse transcriptase (Invitrogen) followed by SYBR Green technology on BioRad CFX96 real-time PCR system (BioRad, Hercules, CA, USA). The PCR primers for the detection of mRNA precursors of NPY, POMC, and AgRP have been published [20].

### In silico analysis of the presence of the H.alvei and E.coli ClpB gene in human gut microbiota

In silico screening was performed against the 9,879,896 microbial genes catalog representative of the human intestinal microbiota (IGC catalog) established from metagenomic assembling of 1267 individuals [17], for details see the supplemental data. For this study, only data from 569 healthy individuals (384 European and 185 Chinese) with their annotated BMI were considered. Ethical approvals for the sample collection and informed consent have been obtained by National ethical committee for all studied groups [17]. The amino acid sequence of the chaperone protein ClpB of *E. coli* K12 (also known as heat shock protein F84.1; NCBI accession number: NP_417083.1) was used as query to screen the metagenomic data. The reference catalog was screened at the protein level using Blastp (v.2.7.1 + ) with an *e*-value threshold of 1E−5 and 40% identity at least over 90% of protein length. Targeted ClpB encoding gene presence and abundance were measured using IGC abundance table established in Li et al. [18].

### Statistical analysis

Data were analyzed using GraphPad Prism 5.02 (GraphPad Software Inc., San Diego, CA, USA). Group differences were compared by the analysis of variance (ANOVA) with Tukey’s post tests or Kruskal–Wallis test followed by Dunn’s post tests according to normality results evaluated by the Kolmogorov-Smirnov test. Individual group differences were analyzed using two-sided Student’s *t*-test or Mann–Whitney’s test. Dynamics of body weight and food intake changes were analyzed by two-way repeated measurments (RM) ANOVA followed by Bonferroni’s post tests. For all tests, *p* < 0.05 was considered statistically significant. Results are shown as mean ± SEM.

## Results

### E.coli K12 native but not the ClpB mutant strain decreases body weight gain in obese mice

**Fig. 1:**
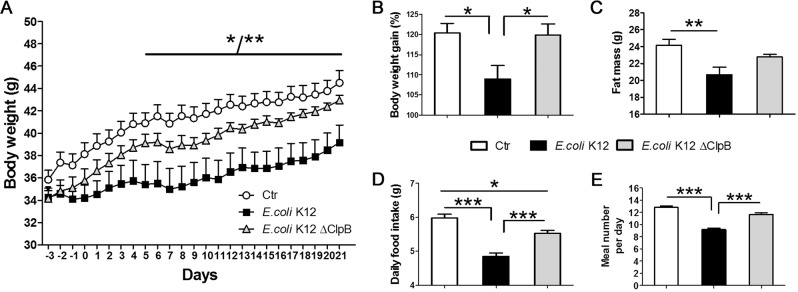
Effects of *E.coli* K12 native and ClpB-deficient strains in *ob/ob* mice. **a** Body weight dynamics. **b** Body weight gain (%). **c** Total fat mass. **d** Daily food intake. **e** Daily meal number. **a** 2-Way RM ANOVA *p* < 0.01, Bonferroni post tests **p < 0.01 for days 6, 8, 11, and 12, other days *p < 0.05. **b** ANOVA *p* < 0.05, Tukey’s post tests *p < 0.05. **c** ANOVA *p* < 0.01, Tukey’s post tests **p < 0.01. **d** ANOVA *p* < 0.0001, Tukey’s post tests ***p < 0.001, *p < 0.05. **e** Kruskal–Wallis *p* < 0.0001, Dunn’s post tests ***p < 0.001.

A decrease in body weight gain was observed after 4 days of treatment by *E.coli* K12 bacteria and persisted throughout the following 16 days of the study (Fig. 1a). Per os administration of mice with the ClpB mutant *E.coli* K12 strain had no significant effect on body weight gain (Fig. 1a, b). Total fat mass was also decreased in mice receiving *E.coli* K12 native but not the ClpB mutant strain (Fig. 1c), the latter was significant when compared directly with the control group (Student’s *t*-test *p* < 0.05). Analysis of feeding behavior revealed that supplementation in *E.coli* K12 reduced the total daily food intake as compared with both control and ClpB-deficient strain-fed mice (Fig. 1d). Decrease in total food intake was due to a decrease in the number of meals (Fig. 1e) without changing the meal size (not shown). A weaker anorexigenic effect was observed in mice supplemented with the ClpB mutant strain (Fig. 1d) which was also due to a decrease in meal number (Student’s *t*-test *p* < 0.01 vs. controls) without affecting meal size (not shown).

### Hafnia alvei HA4597 produces ClpB protein with α-MSH-like epitope

**Fig. 2:**
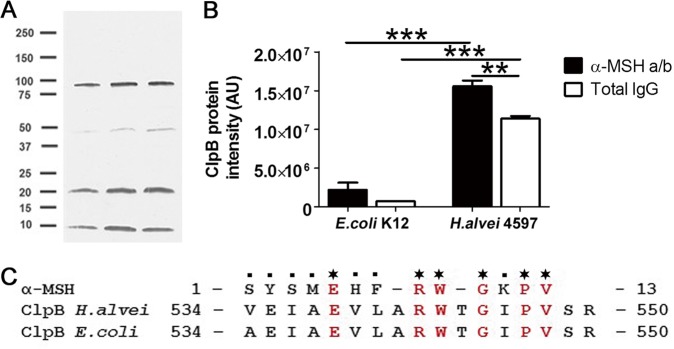
Proteomic analysis of *H.alvei* HA4597 for α-MSH-mimetic proteins. **a** Western blot of *H.alvei* extracted total proteins using anti-α-MSH antibodies (each lane correspond to a different *H.alvei* sample). **b** Intensity of the ClpB protein recovered from *H.alvei* and *E.coli* K12 after immunoprecipitation using rabbit polyclonal α-MSH antibodies or total rabbit IgG and identified by mass spectrometry (AU arbitrary units). **c** Amino acid sequence alignment between the α-MSH peptide and the ClpB proteins from *H.alvei* and *E.coli*. Stars indicate identical amino acids, periods indicate weak similarity <0.5 of Gonnet PAM250 Matrix used in the EMBOS Stretcher program (https://www.ebi.ac.uk/Tools/psa/). **b** ANOVA, *p* < 0.0001, Tukey’s post tests ***p < 0.001, **p < 0.01.

WB on *H.alvei* extracted proteins using anti-α-MSH polyclonal antibodies revealed several bands including one at about 96 KDa corresponding to the molecular weight of the full ClpB molecule, and a further two bands at around 20 and 10 KDa (Fig. 2a).

Using mass spectrometry identification following the immunoprecipitation of *H.alvei* HA4597 and *E.coli* K12 total proteins with an anti-α-MSH polyclonal antibody, 507 ± 70 and 792 ± 28 proteins were respectively recovered and 114 and 96 proteins, respectively, were significantly enriched in samples precipitated by α-MSH antibodies vs. total IgG (data not shown). ClpB was among the recovered proteins in both strains but it was about 10 times more abundant in *H.alvei* HA4597 than in *E.coli* K12 (Fig. 2b). High levels of ClpB recovery by total IgG was expected due to the natural presence of ClpB-reactive IgG in non-immunized animals and humans [12]. Sequence alignments between α-MSH and recovered proteins showed that only the ClpB protein from both *H.alvei* HA4597 and *E.coli* K12 strains displayed the α-MSH-like epitope with the predicted melanocortin-like activity (Fig. 2c). These data were confirmed and further extended by in silico analysis of the bacterial protein reference database (see below).

### Hafnia alvei HA4597 reduces body weight gain and fat mass in ob/ob and HFD obese mice

**Fig. 3:**
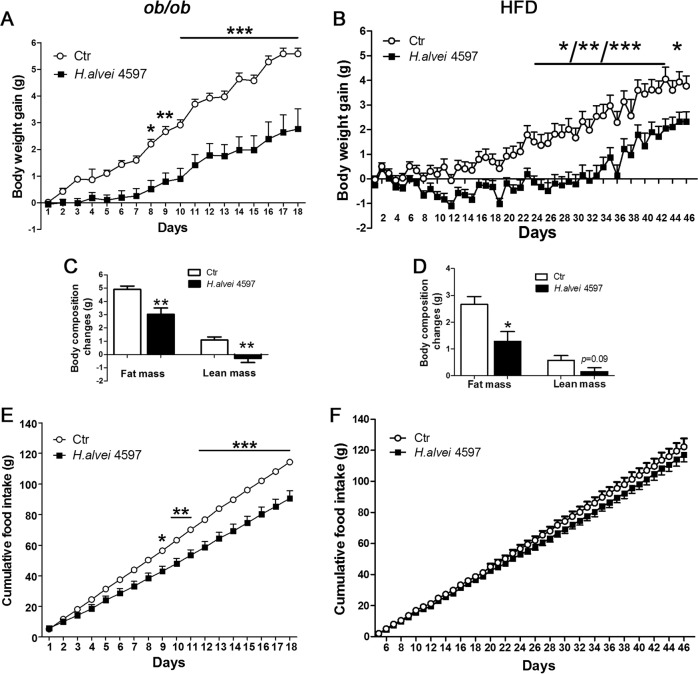
Effects of *H.alvei* HA4597 in *ob/ob* and HFD-fed obese mice on body weight, body composition, and food intake. Body weight dynamics in *ob/ob* (**a**) and in HFD-fed obese mice (**b**). Total fat and lean tissue mass in *ob/ob* (**c**) and in HFD-fed obese mice (**d**). Cumulative food intake in *ob/ob* (**e**) and in HFD-fed obese mice (**f**). Two-way RM ANOVA, **a**, **b** *p* < 0.001, **e** *p* < 0.05, Bonferroni post tests, ***p < 0.001, **p < 0.01, *p < 0.05, **b** *for days 23–25, 38, 41, and 47, **for days 26, 27, 30, 37, 39, 42, and 43, ***for days 28, 29, 31–36, and 40. **c**, **d** Mann–Whitney tests, **p < 0.01, *p < 0.05.

*H.alvei* treatment was not accompanied by any adverse effects. In *ob/ob* mice, body weight gain was significantly lower after 9 days of *H.alvei* HA4597 gavage resulting in a 50.1% decrease at the last day of treatment (Fig. 3a). In the HFD mouse model, prior to *H.alvei* provision, the obesity was induced by HFD (Supplementary Fig. 1). In HFD-fed obese mice, the body weight gain decreased during the *H.alvei* HA4597 treatment as compared with the control group starting from the 23rd day of treatment (Fig. 3b), with the largest difference observed at day 33 corresponding to a 94.7% decrease (0.13 ± 0.41 vs. 2.53 ± 0.43 g). At the last day of treatment this difference decreased to a 38.1% (2.3 ± 0.4 vs. 3.8 ± 0.41 g, Student’s *t*-test *p* < 0.05).

Analysis of the body composition revealed lower gain of fat and loss of lean mass in *H.alvei* HA4597-treated *ob/ob* mice (Fig. 3c), corresponding to a 38.3% and 126.8% decrease, respectively. A decrease of fat mass gain was also observed in the HFD model, corresponding to 51.9%, while a mean decrease of lean mass (72.4%) did not reach significance (Fig. 3d).

### Hafnia alvei HA4597 reduces food intake in ob/ob mice

In the hyperphagic *ob/ob* model, *H.alvei* HA4597 treatment was accompanied by significantly lower cumulative food intake observed from day 9 and resulting in a 20.8% decrease in food intake at the last day of the experiment (Fig. 3e). No significant differences of cumulative food intake were observed between the *H.alvei* HA4597-treated and the control mice in the HFD-induced obesity model (Kruskal–Wallis test *p* = 0.29), although a separation of the cumulative food intake curves was visible between the two groups after 4 weeks of treatment (Fig. 3f).

### H.alvei HA4597 increases ClpB DNA and plasma levels of ClpB protein in obese mice

**Fig. 4:**
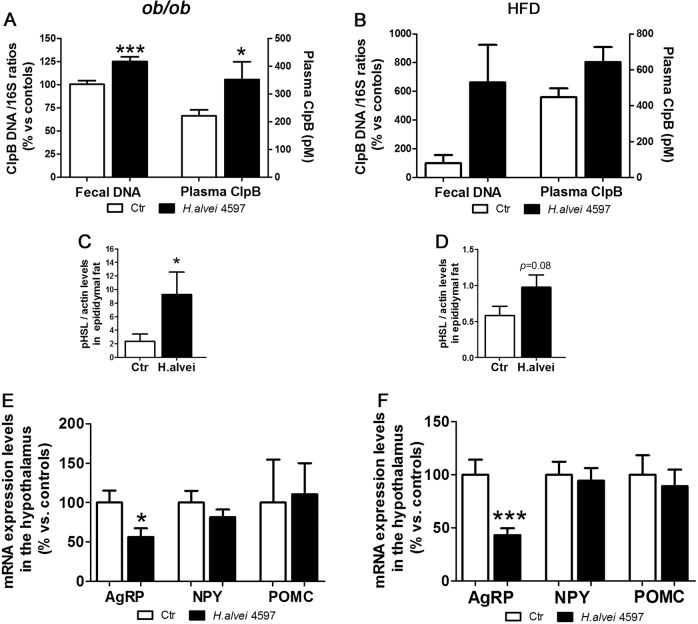
Effects of *H.alvei* HA4597 in *ob/ob* and HFD-fed obese mice on ClpB production, pHSL levels, and hypothalamic neuropeptide mRNA. Levels of ClpB DNA in colonic feces and of ClpB protein in plasma of *ob/ob* (**a**) and of HFD-fed obese mice (**b**). Actin-normalized pHSL levels in the epididymal fat tissue in *ob/ob* (**c**) and in HFD-fed obese mice (**d**). Hypothalamic mRNA expression levels of AgRP, NPY, and POMC in *ob/ob* (**c**) and in HFD-fed obese mice (**d**) relative to controls (100%). **a** Fecal DNA, Student’s *t*-test ***p < 0.001. **a**, **e**, **f** Mann–Whitney tests, *p < 0.05, ***p < 0.001.

*H.alvei* provision increased colonic content of ClpB DNA and plasma concentrations of ClpB protein in *ob/ob* mice (Fig. 4a). Although a tendency of increased ClpB DNA content was observed in HFD obese mice treated by *H.alvei* HA4597 (Mann–Whitney test *p* = 0.09), the plasma concentration of ClpB protein did not change significantly (Mann–Whitney test *p* = 0.49), (Fig. 4b).

### H. alvei HA4597 increases lipolytic marker in obese mice

Using the WB, we found that after 18 days of *H.alvei* treatment in o*b/ob* mice, the pHSL levels were significantly higher than in the control group (Fig. 4c). In the HFD obese mice treated with *H.alvei* a trend of increasing pHSL levels was observed (Mann–Whitney test *p* = 0.08), (Fig. 4d).

### H. alvei HA4597 decreases AgRP mRNA in the hypothalamus of obese mice

We found that in both o*b/ob* and HFD obese mice, *H.alvei* HA4597 treatment was accompanied by lower mRNA expression levels of an orexigenic neuropeptide AgRP (Fig. 4e, f). However, we did not find significant differences in NPY and POMC mRNA expression levels (Fig. 4e, f).

### Enterobacterial ClpB gene is depleted in gut microbiota of obese humans

**Fig. 5:**
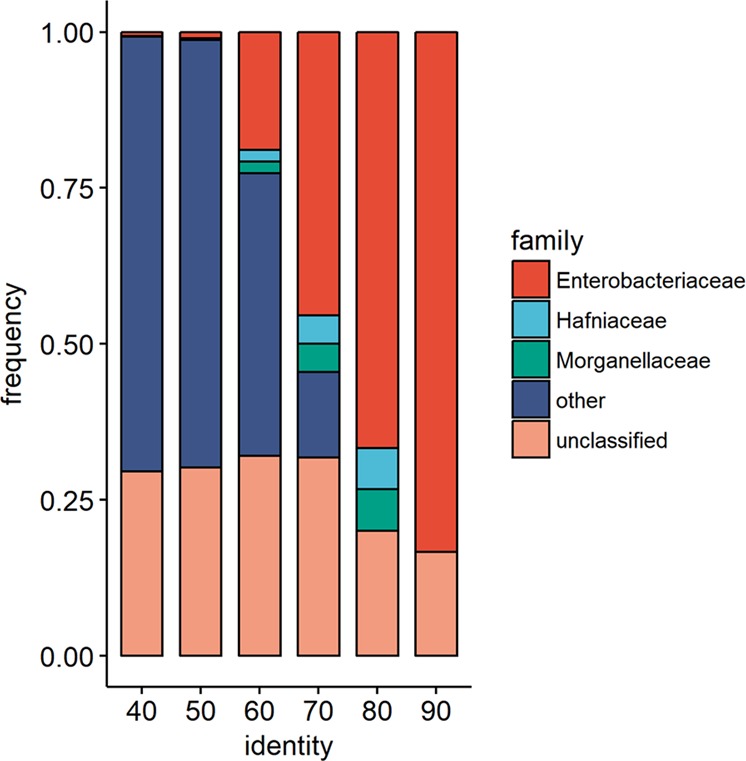
Taxonomic distribution at family level of *E.coli* ClpB homology according to sequence identity (from 40 to 90%). Note that all identified families with more that 80% homology belong to the order of *Enterobacteriales*. Genus *Hafnia* belongs to the new family of *Hafniaceae* and genus *Proteus* to the new family of *Morganellaceae.*

Using Blastp, the 10 M protein reference catalog was interrogated for the *E.coli* K12 ClpB protein sequence. A total of 1527 hits were identified with identity ranging between 40 and 100%. Taxonomic distribution according to sequence identity showed that more than 80% identity was present in the order *Enterobacterales* including families of *Enterobacteriaceae*, *Hafniaceae, Morganellaceae* and in some unclassified microorganisms (Fig. 5). Moreover, the search for the α-MSH-like motif of *E.coli* ClpB, as shown in Fig. 2c, resulted in its detection in 12 annotated genomic records all corresponding to the *Enterobacteriales* order and represented by species of *Escherichia coli, Citrobacter portucalensis, Enterobacter cloacae, Enterobacter xiangfangensis, Klebsiella pneumoniae, Klebsiella michiganensis, Klebsiella oxytoca, Klebsiella aerogenes, Proteus mirabilis, and Hafnia paralvei* (Supplementary Table 1). Of note, there is no specie annotated directly to *Hafnia alvei* in the catalog.

**Fig. 6:**
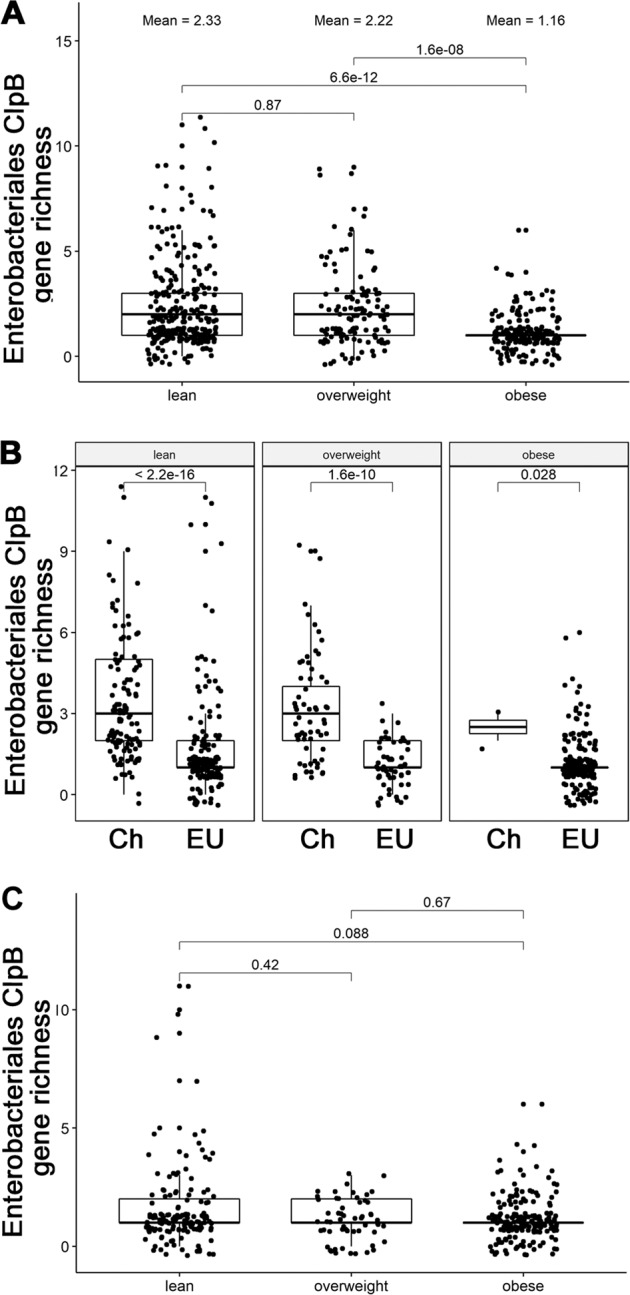
Enterobacteriales ClpB gene richness in human gut microbiota according to BMI status. **a** Total combined cohort. **b** Chinese (Ch) and European (EU) cohorts. **c** European cohort. *P* values for the Mann–Whitney test are shown.

When the *Enterobacterales* ClpB gene species richness were analyzed for their relation to BMI, the microbiota of obese individuals was found to be depleted from these genes as compared with lean and overweight individuals (*p* < 0.0001) but there were no significant differences between the latter groups (Fig. 6a). However, the main contribution of such difference was due to high richness of the *Enterobacterales* ClpB genes in the Chinese cohort as compared with Europeans (Fig. 6b). Nevertheless, it is of interest that mean BMI values in both lean and overweight groups were significantly lower in Chinese vs. European cohorts (21.29 ± 0.15 vs. 22.39 ± 0.14, Mann–Whitney test, *p* < 0.0001 and 26.49 ± 0.1 vs. 27.32 ± 0.2, Mann–Whitney test, *p* < 0.01, respectively). Within the European cohort, only a tendency of increased ClpB gene richness in lean vs. obese group was observed (Fig. 6c). No significant differences were found between three BMI groups within the Chinese cohort, although the obese group was underrepresented having only two individuals (Fig. 6b). To avoid potential bias, Chinese cohort has been excluded in the BMI correlation analysis. Significant negative correlations within the European cohort were found between BMI and abundance of four *Enterobacterales* ClpB species. These included *Enterobacter xiangfangensis*, *Klebsiella michiganensis 1, Klebsiella michiganensis 2,* and *Hafnia paralvei* (Spearman’s rho −0.10, *p* = 0.04 and rho −0.11, *p* = 0.02, rho −0.12, *p* = 0.02, and rho −0.12, *p* = 0.02, respectively, Supplementary Fig. 2). These four species were significantly enriched in lean individuals compared with obese individuals (FDR adjusted *p* = 0.04).

Although *Hafnia alvei* specie was not detected in the IGC reference catalog which lacks its full genomic annotation, the closed phylogenetically *Hafnia paralvei* (msp_1240) was weakly represented. Only 13 healthy individuals harbored this specie in their gut microbiota. Using an alternative bioinformatics approach, blasting of the *H.alvei* HA4597 specie genome resulted in its detection in metagenome of eight healthy humans i.e., it represents a relatively rare commensal gut specie.

## Discussion

Bacteria of the genus *Hafnia* were identified and named in 1954 by Danish microbiologist Møller after the Latin name for Copenhagen [21]. The food origin of *H.alvei* was documented by its isolation from raw milk and subsequently from cheese and is considered as part of normal milk microbiota [22, 23]. High numbers of *H.alvei* have been reported in Camembert cheese, up to 10^8^ of viable bacteria per g [24]. Since the nineties, *H.alvei* has been added to milk during the manufacturing of soft cheese due to its beneficial cheese ripening properties. As a result, these bacteria have been consumed naturally and in high amounts for decades by the general population. There have been no adverse effects identified in immunocompetent individuals associated with cheese-derived *H.alvei* intake, therefore, justifying it as a safe food-grade bacteria annotated by The International Dairy Federation for Microbial Food Cultures [25]. Furthermore, its random presence in the gut microbiota of healthy humans justifies the appellation of *H.alvei* as a gut commensal.

It is of relevance for the present study to note that *H.alvei* is the only commensal specie from the *Enterobacteriales* order to date to carry a food-grade status. In fact, although some other species such as *E.coli* or *Enterobacter* are common members of the human gut microbiota, they do not have food-grade status and their presence in food products is considered as a marker of fecal microbial contamination [26]. Therefore, the aforementioned food-grade properties of *H.alvei* as well as the expression of the ClpB gene with the same α-MSH-like motif as in *E.coli* served for its selection and validation as a potential probiotic strain for appetite and body weight management in the present study.

Anti-obesity probiotic should be able to interfere with at least two major pro-obesity factors such as hyperphagia and fat tissue accumulation [27]. In this aspect, activation of specific melanocortin receptors (MCR) by α-MSH represents a well-established pharmacological target triggering both anorexigenic and lipolytic effects [28]. Indeed, a recent report showed a marked anti-obesity effect of an α-MSH synthetic peptide analog setmelanotide in two patients with congenital obesity caused by POMC deficiency [29]. Thus, as long as safe and efficient pharmacological anti-obesity compounds continue being developed, a probiotic with natural α-MSH mimetic properties may become an option for obesity and overweight prevention or treatment.

In the present study, we confirmed that the α-MSH-like motif, initially described in *E.coli* K12 ClpB is conserved in the *Enterobacteriales* order including the *Hafnia* genus and that the *H.alvei* HA4597 strain synthetizes the α-MSH-mimetic ClpB protein. The recovery of some other bacterial proteins by immunoprecipitation with α-MSH antibodies was due to their binding α-MSH-non related or possibly shorter α-MSH-like motifs which did not contain the melanocortin core sequence i.e., they cannot *a priori* activate the MCR. For instance, a mimicry of the α-MSH N-terminal was previously identified in the elongation factor-G of *E.coli* by the Roth’s group using corticotropin antibodies [30]. We were also able to detect this protein in *H.alvei* after precipitation with an α-MSH antibody but it was about 600 times less abundant than the ClpB (data not shown). We also showed for the first time that *per os* administration of a laboratory *E.coli* strain from the same with *H.alvei Enterobacteriales* order in *ob/ob* mice decreases their food intake, fat mass and body weight gain and that ClpB was the main active substance responsible for these effects. Although the exact mechanisms of action of ClpB on the regulation of appetite and energy metabolism remain to be established, the α-MSH-mimetic properties may play a key role because the appetite-reducing effects of traditional probiotics *Lactobacillus* and *Bifidobacterium*, known to express ClpB-like proteins with no homology to α-MSH [31], have not been reported in either animal or human studies [9]. With regard to the possible ClpB action sites, it activated the hypothalamic anorexigenic POMC neurons [18] and a recent report showed the capacity of ClpB to stimulate secretion of peptide YY, a satiety hormone from the gut [32, 33]. The underlying receptors and intracellular pathways still need to be determined, but the ability of the α-MSH-like motif of ClpB to activate cAMP release by type one MCR has been shown [34] pointing to the spatial complementarity between this ClpB peptide fragment and the MCRs.

Our data revealed that *H.alvei* HA4597 displays the desired probiotic properties of an anti-obesity or weight loss supplement i.e., produce anorexigenic and lipolytic effects in hyperphagic *ob/ob* mice resulting in decreased fat mass and body weight gain. The body weight and fat tissue lowering effects were also observed in HFD obese mice, although without significant anorexigenic and lipolytic actions. Considering low grade obesity and hypophagia in the HFD obesity model, moderate anorexigenic and lipolytic actions of *H.alvei* HA4597 may cumulate to reduce total adiposity. In fact, decreased levels of AgRP mRNA expression in the hypothalamus were found in both *ob/ob* and HFD mice indicating inhibition of the brain orexigenic circuitry by *H.alvei*. AgRP neurons are known to be inhibited by PYY [35] and, hence, may transmit intestinal satietogenic effects triggered by *H.alvei* ClpB. Indeed, intestinal epithelium including the enteroendocrine cells was shown to express MCR [36].

Finally, the low abundance of ClpB gene expressing *Enterobacterales* species found in the microbiota of obese subjects in the present in silico analysis may indicate insufficient anorexigenic signaling from the gut microbiota to the host, further providing the rationale for supplementation of commensal bacteria expressing the ClpB protein with an α-MSH-like motif. These results are in agreement with previous reports showing a low prevalence of *Enterobacteriaceae* in obese individuals [37]. Thus, our study has validated *H.alvei* HA4597 food-grade bacterial strain as a new potential anti-obesity and anti-overweight probiotic because the desired effects of lowering appetite and/or body weight have been obtained in two mouse models of obesity and overweight.

## Supplementary information

Supplementary Table 1

Supplementary Table 2

Supplementary data

## References

[1] Rosenbaum M, Knight R, Leibel RL (2015). The gut microbiota in human energy homeostasis and obesity. Trends Endocrinol Metab..

[2] Sender R, Fuchs S, Milo R (2016). Revised estimates for the number of human and bacteria cells in the body. PLoS Biol.

[3] Yatsunenko T, Rey FE, Manary MJ, Trehan I, Dominguez-Bello MG, Contreras M (2012). Human gut microbiome viewed across age and geography. Nature.

[4] Bolnick DI, Snowberg LK, Hirsch PE, Lauber CL, Org E, Parks B (2014). Individual diet has sex-dependent effects on vertebrate gut microbiota. Nat Commun.

[5] Cotillard A, Kennedy SP, Kong LC, Prifti E, Pons N, Le Chatelier E (2013). Dietary intervention impact on gut microbial gene richness. Nature.

[6] Turnbaugh PJ, Ley RE, Mahowald MA, Magrini V, Mardis ER, Gordon JI (2006). An obesity-associated gut microbiome with increased capacity for energy harvest. Nature.

[7] Fetissov SO (2017). Role of the gut microbiota in host appetite control: bacterial growth to animal feeding behaviour. Nat Rev Endocrinol.

[8] Muscogiuri G, Cantone E, Cassarano S, Tuccinardi D, Barrea L, Savastano S, et al. Gut microbiota: a new path to treat obesity. Int J Obes Suppl. 2019. 10.1038/s41367-019-0011-7. 10.1038/s41367-019-0011-7 PMC6683132 31391921

[9] Kobyliak N, Conte C, Cammarota G, Haley AP, Styriak I, Gaspar L (2016). Probiotics in prevention and treatment of obesity: a critical view. Nutr Metab (Lond).

[10] Crovesy L, Ostrowski M, Ferreira DMTP, Rosado EL, Soares-Mota M (2017). Effect of Lactobacillus on body weight and body fat in overweight subjects: a systematic review of randomized controlled clinical trials. Int J Obes.

[11] Cani PD, de Vos WM. Next-generation beneficial microbes: the case of akkermansia muciniphila. Front Microbiol. 2017;8:1765. 10.3389/fmicb.2017.01765 PMC5614963 29018410

[12] Tennoune N, Chan P, Breton J, Legrand R, Chabane YN, Akkermann K (2014). Bacterial ClpB heat-shock protein, an antigen-mimetic of the anorexigenic peptide [alpha]-MSH, at the origin of eating disorders. Transl Psychiatry.

[13] Yaswen L, Diehl N, Brennan MB, Hochgeschwender U (1999). Obesity in the mouse model of pro-opiomelanocortin deficiency responds to peripheral melanocortin. Nat Med.

[14] Krude H, Biebermann H, Luck W, Horn R, Brabant G, Gruters A (1998). Severe early-onset obesity, adrenal insufficiency and red hair pigmentation caused by POMC mutations in humans. Nat Genet.

[15] Farooqi IS, Keogh JM, Yeo GSH, Lank EJ, Cheetham T, O'Rahilly S (2003). Clinical spectrum of obesity and mutations in the melanocortin 4 receptor gene. N Engl J Med.

[16] Adeolu M, Alnajar S, Naushad S, S. Gupta R (2016). Genome-based phylogeny and taxonomy of the ‘Enterobacteriales’: proposal for Enterobacterales ord. nov. divided into the families Enterobacteriaceae, Erwiniaceae fam. nov., Pectobacteriaceae fam. nov., Yersiniaceae fam. nov., Hafniaceae fam. nov., Morganellaceae fam. nov., and Budviciaceae fam. nov. Int J Syst Evol Microbiol.

[17] Li J, Jia H, Cai X, Zhong H, Feng Q, Sunagawa S (2014). An integrated catalog of reference genes in the human gut microbiome. Nat Biotechnol.

[18] Breton J, Tennoune N, Lucas N, François M, Legrand R, Jacquemot J (2016). Gut commensal *E.coli* proteins activate host satiety pathways following nutrient-induced bacterial growth. Cell Metab.

[19] Turner S, Pryer KM, Miao VPW, Palmer JD (1999). Investigating deep phylogenetic relationships among cyanobacteria and plastids by small subunit rRNA sequence analysis1. J Eukaryotic Microbiol.

[20] Hamze Sinno M, Do Rego JC, Coëffier M, Bole-Feysot C, Ducrotte P, Gilbert D (2009). Regulation of feeding and anxiety by α-MSH reactive autoantibodies. Psychoneuroendocrinology.

[21] Moller V (1954). Distribution of amino acid decarboxylases in Enterobacteriaceae. Acta Pathol Microbiol Scand.

[22] Gaya P, Medina M, Nuntez M (1987). Enterobacteriaceae, coliforms, faecal coliforms and salmonellas in raw ewes'milk. J Appl Bacteriol.

[23] Tornadijo E, Fresno JM, Carballo J, Martín-Sarmiento R (1993). Study of Enterobacteriaceae throughout the manufacturing and ripening of hard goats' cheese. J Appl Bacteriol.

[24] Richard J, Zadi H (1983). Inventaire de la flore bactérienne dominante des Camemberts fabriqués avec lait cru. Le Lait, INRA Ed.

[25] Federation ID (2012). Safety demonstration of microbial food cultures (MFC) in fermented food products (Annex 4). Bull Int Dairy Fed.

[26] Mogren L, Windstam S, Boqvist S, Vågsholm I, Söderqvist K, Rosberg AK, et al. The hurdle approach—a holistic concept for controlling food safety risks associated with pathogenic bacterial contamination of leafy green vegetables. A Review. Front Microbiol. 2018;9:1965. 10.3389/fmicb.2018.01965 PMC6117429 30197634

[27] Richard D (2015). Cognitive and autonomic determinants of energy homeostasis in obesity. Nat Rev Endocrinol.

[28] Anderson EJP, Çakir I, Carrington SJ, Cone RD, Ghamari-Langroudi M, Gillyard T (2016). 60 YEARS OF POMC: regulation of feeding and energy homeostasis by α-MSH. J Mol Endocrinol.

[29] Kühnen P, Clément K, Wiegand S, Blankenstein O, Gottesdiener K, Martini LL (2016). Proopiomelanocortin deficiency treated with a melanocortin-4 receptor agonist. N Engl J Med.

[30] Qiang X, Liotta AS, Shiloach J, Gutierrez JC, Wang H, Ochani M (2017). New melanocortin-like peptide of E. coli can suppress inflammation via the mammalian melanocortin-1 receptor (MC1R): possible endocrine-like function for microbes of the gut. NPJ Biofilms Microb.

[31] Fetissov SO, Legrand R, Lucas N (2019). Bacterial protein mimetic of peptide hormone as a new class of protein-based drugs. Curr Med Chem.

[32] Dominique M, Breton J, Guérin C, Bole-Feysot C, Lambert G, Déchelotte P (2019). Effects of macronutrients on the in vitro production of ClpB, a bacterial mimetic protein of α-MSH and its possible role in the satiety signaling. Nutrients.

[33] Cox HM, Tough IR, Woolston A-M, Zhang L, Nguyen AD, Sainsbury A (2010). Peptide YY is critical for acylethanolamine receptor Gpr119-induced activation of gastrointestinal mucosal responses. Cell Metab.

[34] Ericson MD, Schnell SM, Freeman KT, Haskell-Luevano C (2015). A fragment of the Escherichia coli ClpB heat-shock protein is a micromolar melanocortin 1 receptor agonist. Bioorg Med Chem Lett.

[35] Batterham RL, Cowley MA, Small CJ, Herzog H, Cohen MA, Dakin CL (2002). Gut hormone PYY(3-36) physiologically inhibits food intake. Nature.

[36] Panaro Brandon L, Tough Iain R, Engelstoft Maja S, Matthews Robert T, Digby Gregory J, Møller Cathrine L (2014). The melanocortin-4 receptor is expressed in enteroendocrine L cells and regulates the release of peptide YY and glucagon-like peptide 1 in vivo. Cell Metab.

[37] Million M, Angelakis E, Maraninchi M, Henry M, Giorgi R, Valero R (2013). Correlation between body mass index and gut concentrations of Lactobacillus reuteri, Bifidobacterium animalis, Methanobrevibacter smithii and Escherichia coli. Int J Obes.

