# Supplementary material for: Commensal Hafnia alvei strain reduces food intake and fat mass in obese mice—a new potential probiotic for appetite and body weight management
Source: Int J Obes (Lond). 2020 Jan 7;44(5):1041–51. doi: 10.1038/s41366-019-0515-9 (PMC7188665; doi:10.1038/s41366-019-0515-9)
Supplement: Supplementary file 1 — Supplementary Table 1 [file 41366_2019_515_MOESM1_ESM.docx]

| **gene reference** | **% identity with *E.coli* K12 ClpB protein** | **gene length** | **mgs** | **species** | **genus** | **family** | **order** | **α-MSH like motif** |
| --- | --- | --- | --- | --- | --- | --- | --- | --- |
| MH0383_GL0114331 | 100 | 857 | msp_0005 | Escherichia coli | Escherichia | Enterobacteriaceae | Enterobacterales | YES |
| 469595,CSAG_02385 | 96,849 | 857 | msp_0666 | Citrobacter portucalensis | Citrobacter | Enterobacteriaceae | Enterobacterales | YES |
| 290338,CKO_03914 | 96,733 | 857 |  |  |  |  |  | YES |
| T2D-108A_GL0186653 | 96,383 | 857 | msp_1254 | Enterobacter cloacae | Enterobacter | Enterobacteriaceae | Enterobacterales | YES |
| 888063,HMPREF9086_3481 | 96,266 | 857 | msp_0123 | Enterobacter xiangfangensis | Enterobacter | Enterobacteriaceae | Enterobacterales | YES |
| SZEY-40A_GL0050436 | 96,033 | 857 | msp_0205 | Enterobacter cloacae complex 'Hoffmann cluster IV' | Enterobacter | Enterobacteriaceae | Enterobacterales | YES |
| 1045856,EcWSU1_03401 | 95,799 | 857 |  |  |  |  |  | YES |
| 484021,KP1_4170 | 94,982 | 857 | msp_0028 | Klebsiella pneumoniae | Klebsiella | Enterobacteriaceae | Enterobacterales | YES |
| 1006551,KOX_00005 | 94,749 | 857 | msp_0597 | Klebsiella michiganensis 1 | Klebsiella | Enterobacteriaceae | Enterobacterales | YES |
| MH0260_GL0007484 | 94,516 | 857 | msp_0898 | Klebsiella michiganensis 2 | Klebsiella | Enterobacteriaceae | Enterobacterales | YES |
| MH0277_GL0047440 | 94,417 | 806 | msp_0142 | Klebsiella oxytoca | Klebsiella | Enterobacteriaceae | Enterobacterales | YES |
| NLM027_GL0023343 | 94,399 | 857 | msp_0156 | Klebsiella aerogenes | Klebsiella | Enterobacteriaceae | Enterobacterales | YES |
| 469613,HMPREF0864_03166 | 86,581 | 857 | msp_1240 | Hafnia paralvei | Hafnia | Hafniaceae | Enterobacterales | YES |
| V1,CD3-0-PT_GL0038490 | 83,664 | 857 | msp_0221 | Proteus mirabilis | Proteus | Morganellaceae | Enterobacterales | YES |
| V1,UC39-4_GL0123735 | 83,664 | 857 |  |  |  |  |  | YES |

**Supplementary table 1.** Results of the search the 10M bacterial protein reference catalog for the presence of bacteria expressing proteins with specific α-MSH-like motif of *E.coli* ClpB.
