# Supplementary material for: Commensal Hafnia alvei strain reduces food intake and fat mass in obese mice—a new potential probiotic for appetite and body weight management
Source: Int J Obes (Lond). 2020 Jan 7;44(5):1041–51. doi: 10.1038/s41366-019-0515-9 (PMC7188665; doi:10.1038/s41366-019-0515-9)
Supplement: Supplementary file 2 — Supplementary Table 2 [file 41366_2019_515_MOESM2_ESM.docx]

| **mgs** | **species** | **genus** | **family** | **order** | **bmi_rho** | **bmi_rho_p** | **prevalence** |
| --- | --- | --- | --- | --- | --- | --- | --- |
| msp_0005 | Escherichia coli | Escherichia | Enterobacteriaceae | Enterobacterales | -0,083 | 0,101 | 308 |
| msp_0666 | Citrobacter portucalensis | Citrobacter | Enterobacteriaceae | Enterobacterales | -0,014 | 0,771 | 24 |
| msp_1254 | Enterobacter cloacae | Enterobacter | Enterobacteriaceae | Enterobacterales | -0,093 | 0,067 | 5 |
| msp_0123 | Enterobacter xiangfangensis | Enterobacter | Enterobacteriaceae | Enterobacterales | -0,101 | **0,047** | 36 |
| msp_0205 | Enterobacter cloacae complex 'Hoffmann cluster IV' | Enterobacter | Enterobacteriaceae | Enterobacterales | -0,074 | 0,143 | 9 |
| msp_0028 | Klebsiella pneumoniae | Klebsiella | Enterobacteriaceae | Enterobacterales | -0,024 | 0,632 | 57 |
| msp_0597 | Klebsiella michiganensis 1 | Klebsiella | Enterobacteriaceae | Enterobacterales | -0,114 | **0,024** | 13 |
| msp_0898 | Klebsiella michiganensis 2 | Klebsiella | Enterobacteriaceae | Enterobacterales | -0,118 | **0,020** | 14 |
| msp_0142 | Klebsiella oxytoca | Klebsiella | Enterobacteriaceae | Enterobacterales | -0,051 | 0,311 | 9 |
| msp_0156 | Klebsiella aerogenes | Klebsiella | Enterobacteriaceae | Enterobacterales | -0,004 | 0,933 | 6 |
| msp_1240 | Hafnia paralvei | Hafnia | Hafniaceae | Enterobacterales | -0,119 | **0,018** | 11 |
| msp_0221 | Proteus mirabilis | Proteus | Morganellaceae | Enterobacterales | -0,053 | 0,297 | 7 |

**Supplementary table 2. Correlations between the abundance of Enterobacterales ClpB species and BMI.**
