# Supplementary material for: Commensal Hafnia alvei strain reduces food intake and fat mass in obese mice—a new potential probiotic for appetite and body weight management
Source: Int J Obes (Lond). 2020 Jan 7;44(5):1041–51. doi: 10.1038/s41366-019-0515-9 (PMC7188665; doi:10.1038/s41366-019-0515-9)
Supplement: Supplementary file 3 — Supplementary data [file 41366_2019_515_MOESM3_ESM.docx]

**Supplementary data**

**Materials and methods**

**1. Identification and quantification of proteins immunoprecipitated by α-MSH antibodies in *E.coli* and *H.alv*ei strain lysates using hyper reaction monitoring (HRM) mass spectrometry**

**(by Biognosys AG, Schlieren, Switzerland).**

*Bacterial protein extraction*

Total proteins from *E.coli* K12 and *H.alvei* 4597 (manufactured for TargEDys SA by Biodis, Noyant, France) were extracted using 500 μl of lysis buffer for 100 mg of freeze-dried material (100 μL buffer AX2, 2 μL dithiothreitol 100 mM, 50 μL NP40 1%, 1 μL protease inhibitors P8340 1X, 2 μL phosphatase inhibitors P2850 1X, for H2O 200 μL). After centrifugation at 15 000 g for 5 min at 4 °C, the supernatant containing the proteins was collected. Total protein concentrations were determined using a BCA kit in 96 well plates according to the supplier instructions (Thermo Fisher Scientific, MA, USA).

*Sample preparation*

For the immunoprecipitation, 150 μg of rabbit total IgG (Sigma-Aldrich, Germany) and polyclonal rabbit anti-α-MSH antibody (Delphi Genetics, Belgium) were purified using the AbPureAntibody Concentration and Clean-Up Kit (InnovaBiosciences, Cambridge, UK) and bound to magnetic beads using the Magnetic Conjugation Kit (InnovaBiosciences) according to the manufacturer’s instructions. Bacterial lysates were diluted at 5 μg/μl in wash buffer 1 (10 mM HEPES, 10 mM KCl, 0.1 mM EDTA, 0.25 % NP-40, pH 7.5). 200 μg of proteins per lysate were incubated with 5 μg of bound antibodies for 1 h at 4°C in an end-over-end shaker. Using a magnetic rack, beads were washed and transferred to wash buffer 2 (10 mM HEPES, 10 mM KCl, 0.1 mM EDTA, pH 7.5). Precipitated proteins were eluted using 3 x 50 μl of elution buffer (6 M urea, 0.1 mM ABC) and reduced/alkylated using Biognosys’ Reduction/Alkylation Solution for 1 h at 37 °C. Digestion was carried out using 1 μg trypsin (Promega, France) overnight at 37 °C. Then, Samples were desalted using C18 UltraMicroSpincolumns (The Nest Group, MA, USA) according to the manufacturer’s instructions and dried down using a SpeedVacsystem. Peptides were resuspended in 15 μl LC solvent A (1 % acetonitrile, 0.1 % formic acid; FA) and spiked with Biognosys’ iRT kit calibration peptides prior to mass spectrometric analyses.

*DIA LC-MS/MS for spectral library generation*

For the data independent acquisition (DIA) LC-MS/MS measurements, samples were pooled according to the condition group and 7 μl of resolved peptides were injected into an in-house packed C18 column (Dr. MaischReproSilPur, 1.9 μm particle size, 120 Å pore size; 75 μm inner diameter, 50 cm length, New Objective) on an Easy nLC1200 nano-liquid chromatography system connected to a Fusion LumosTribridmass spectrometer equipped with a standard nano-electrospray source (Thermo Fisher Scientific). LC solvents were A: 1 % acetonitrile in water with 0.1 % FA; B: 15 % water in acetonitrile with 0.1 % FA. The nonlinear LC gradient was 1-52 % solvent B in 1 h followed by 52-90 % B in 10 s, 90 % B for 10 min, 90 % -1 % B in 10 s and 1 % B for 5 min. A modified top speed method (3 s cycle time) from Herbert et al. 2014 was used.

*DIA LC-MS/MS database search*

The mass spectrometric data was analyzed using Pulsar software (Biognosys, Switzerland), the false discovery rate of the peptide and protein level was set to 1%. Data was searched against an E.coli (UniProt TrEMBL, 83333) or *H.alvei* (UniProt TrEMBL, 13337) protein fasta database. The HRM measurements analyzed with Spectronaut were normalized using local regression normalization (Calllister SJ et al. 2006) Distance in the heat maps was calculated using the “manhattan” method, the clustering using “ward.D” for both axis. General plotting was done in R using ggplot2package.

*Statistical analysis*

After immunoprecipitation with the anti-α-MSH antibody only proteins which have a q-value below 0.001 and an average log2 fold change above 0.4 were considered in the study. Relative quantifications of commonly identified proteins in E.coli and H.alvei were expressed as mean ± standard error of means and analyzed using GraphPad Prism 5.0 (GraphPad Software Inc., CA, USA). According to normality results evaluated by the D’Agostino-Pearson test, individual differences were analyzed using the Student t-test or Mann-Whitney test with a p-value<0.05 considered as statistically significant.

**2. Metagenomic data mining for Hafnia alvei specie and ClpB genes screening in context of BMI and obesity (by INRA Metagenopolis).**

*Reference gene catalog*

*In silico* screening was performed against the 9,879,896 (10M) microbial genes catalog representative of the human intestinal microbiota (IGC catalog). This gene catalog was established in the frame of the MetaHIT European project from sequenced DNA sample assembling of 1267 individuals (1).

The reference gene catalog is structured into 1661 metagenomic species (MGS) using MSP (Metagenomic Specie Pangenome) Miner software (2).

*Reference gene catalog screening*

The reference catalog was screened at the protein level using Blastp (v.2.7.1+) with an e-value threshold of 1E-5. Only hits with at least 40% identity over 90% of protein length were considered for further analysis.

Amino acid sequence of chaperone protein ClpB of *E. coli* K12 (also known as heat shock protein F84; NCBI accession number: NP_417083.1) was used as query to screen metagenomic data. Length of this ClpB protein is 857 amino acids. Specific amino acid motif of ClpB proteins were searched for in hits using regular expression through Unix tools such as exact motif AEIAEVLARWTGIPV and degenerated motif E.{,3}RW.?G.PV (taking into account conserved amino acids and using regular expression) were searched for.

*Gene and MGS abundance tables*

Normalized gene abundance table for the 1267 individuals was downloaded from <http://meta.genomics.cn/meta/dataTools> (1). MGS signal among samples was computed as the mean signal of 50 core genes as described in Plaza Onate et al. (2). MGS frequency profile table was constructed using the MGS signals and after normalization (sum of the MGS frequency of a sample = 1).

*Phenotypic data*

From the 1267 individuals integrated in IGC data (1), 139 individuals were discarded as they were not accompanied by phenotypic data. 7 others individuals were discarded as their phenotypic status were ambiguous. Others were removed because duplicated (time-points for same individuals). For remaining 1001 individuals (European and Chinese), the searched data i.e. BMI and clinical status i.e. healthy or obese were available and only 569 healthy individuals were analyzed (384 European and 185 Chinese). To avoid confounding factor (in particular enrichment of *Enterobacteriaceae* family in Chinese sample (1), only 384 healthy European subjects were considered for the BMI correlation analysis. To note that a BMI status (obese/lean/overweight) was determined as defined in Le Chatelier et al (3). Individuals with BMI>=30 are denoted as obese and individuals with BMI<25 are denoted as lean. Other individuals are considered as overweighed.

*Statistical analysis*

Statistical analysis were performed using R software and particularly momr package dedicated to the analysis of large quantitative metagenomics datasets (Prifti E and Le Chatelier. momr R package. https://cran.r-project.org/web/packages/momr/index.html)

*References*

1. Li J, Jia H, Cai X, Zhong H, Feng Q, Sunagawa S, et al. An integrated catalog of reference genes in the human gut microbiome. Nature Biotechnology. 2014;32:834.

2. Plaza Oñate F, Le Chatelier E, Almeida M, Cervino ACL, Gauthier F, Magoulès F, et al. MSPminer: abundance-based reconstitution of microbial pan-genomes from shotgun metagenomic data. Bioinformatics. 2018;35(9):1544-52.

3. Le Chatelier E, Nielsen T, Qin J, Prifti E, Hildebrand F, Falony G, et al. Richness of human gut microbiome correlates with metabolic markers. Nature. 2013;500(7464):541-6.


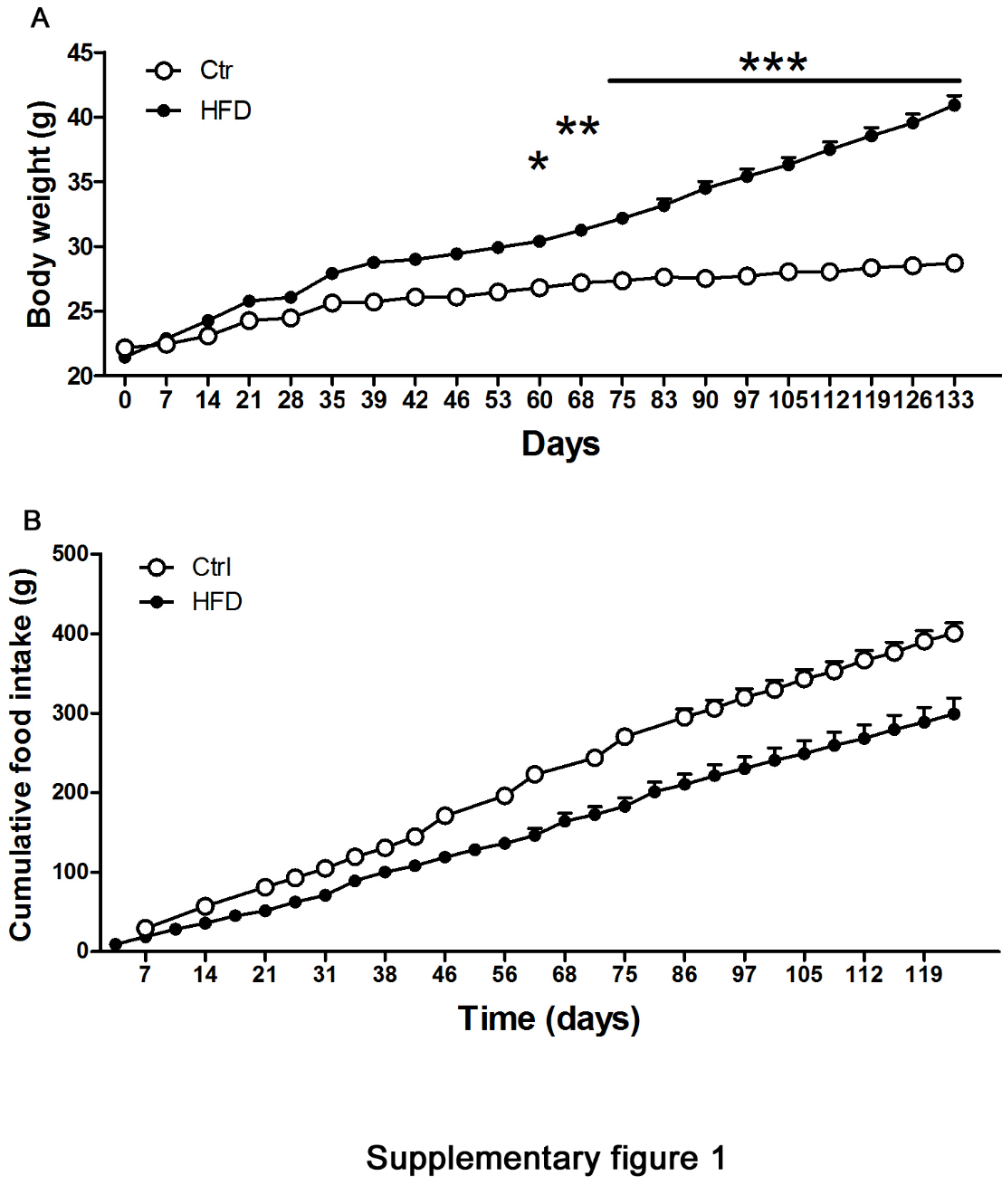


**Supplementary Figure 1. A.** Body weight and **B**. Cumulative food intake during obesity induction in mice fed HFD. **A**. Two-way RM ANOVA p<0.0001, Bonferroni post-tests, ***p<0.001, **p<0.01, *p<0.05.
